# Supplementary material for: TGFβ determines epithelial tissue spacing by regulating mesenchymal condensation
Source: bioRxiv. 2026 Mar 18:2026.03.16.712215. Preprint. [Version 1] doi: 10.64898/2026.03.16.712215 (PMC13015280; doi:10.64898/2026.03.16.712215)
Supplement: Supplement 1 [file NIHPP2026.03.16.712215v1-supplement-1.pdf]

bioRxiv preprint doi: https://doi.org/10.64898/2026.03.16.712215; this version posted March 18, 2026. The copyright holder for this preprint (which was not certified by peer review) is the author/funder, who has granted bioRxiv a license to display the preprint in perpetuity. It is made available under aCC-BY 4.0 International license.

Supplementary Figure Legends

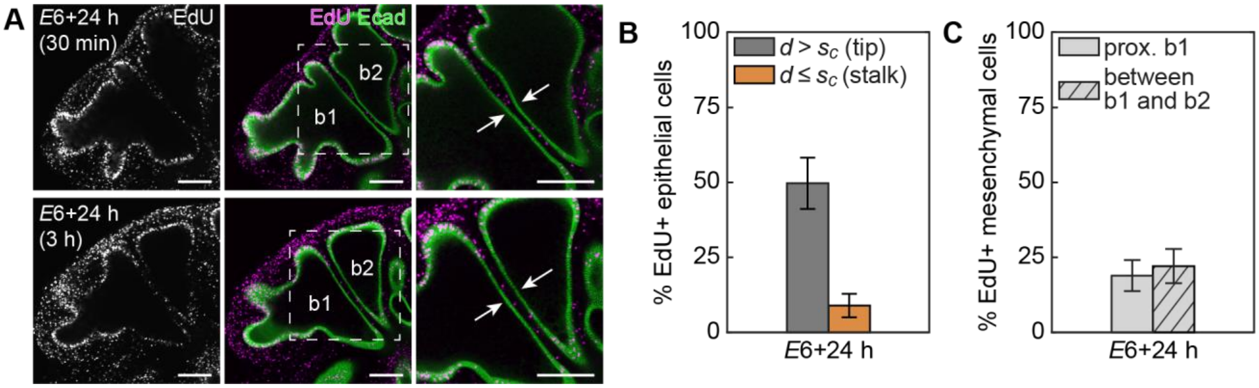

Fig. S1. EdU analysis in the epithelium and mesenchyme of cultured lung explants – related to Fig. 1. (**A**) Fluorescence images of EdU incorporation (magenta) in embryonic chicken lung explants counterstained for E-cadherin (green), with either 30 min or 3 hours of EdU pulse. Scale bars, 100 µm. Graph of percentage of EdU-positive cells in the (**B**) epithelium and (**C**) mesenchyme of cultured lung explants. In panel (**C**), solid and hatched bars denote percentage of EdU-positive mesenchymal cells proximal to b1 and in between b1 and b2, respectively.

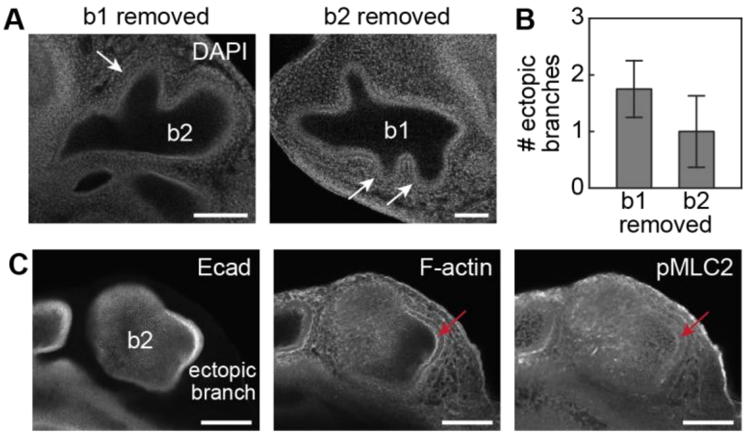

Fig. S2. Analysis of apical constriction in ectopic branches – related to Fig. 2. (**A**) Fluorescence images of nuclei in lung explants after surgical removal of b1 or b2, focused on the remaining neighboring branch. Scale bars, 100 µm. (**B**) Graph showing number of ectopic branches that form in the remaining neighboring branch after surgical removal of b1 (i) or b2 (ii). Shown are average and s.d. of at least 4 lungs per condition. (**C**) Confocal section of an ectopic branch stained for F-actin and pMLC2 lacks noticeable signal on the apical side. Scale bars, 100 µm.

bioRxiv preprint doi: https://doi.org/10.64898/2026.03.16.712215; this version posted March 18, 2026. The copyright holder for this preprint (which was not certified by peer review) is the author/funder, who has granted bioRxiv a license to display the preprint in perpetuity. It is made available under aCC-BY 4.0 International license.

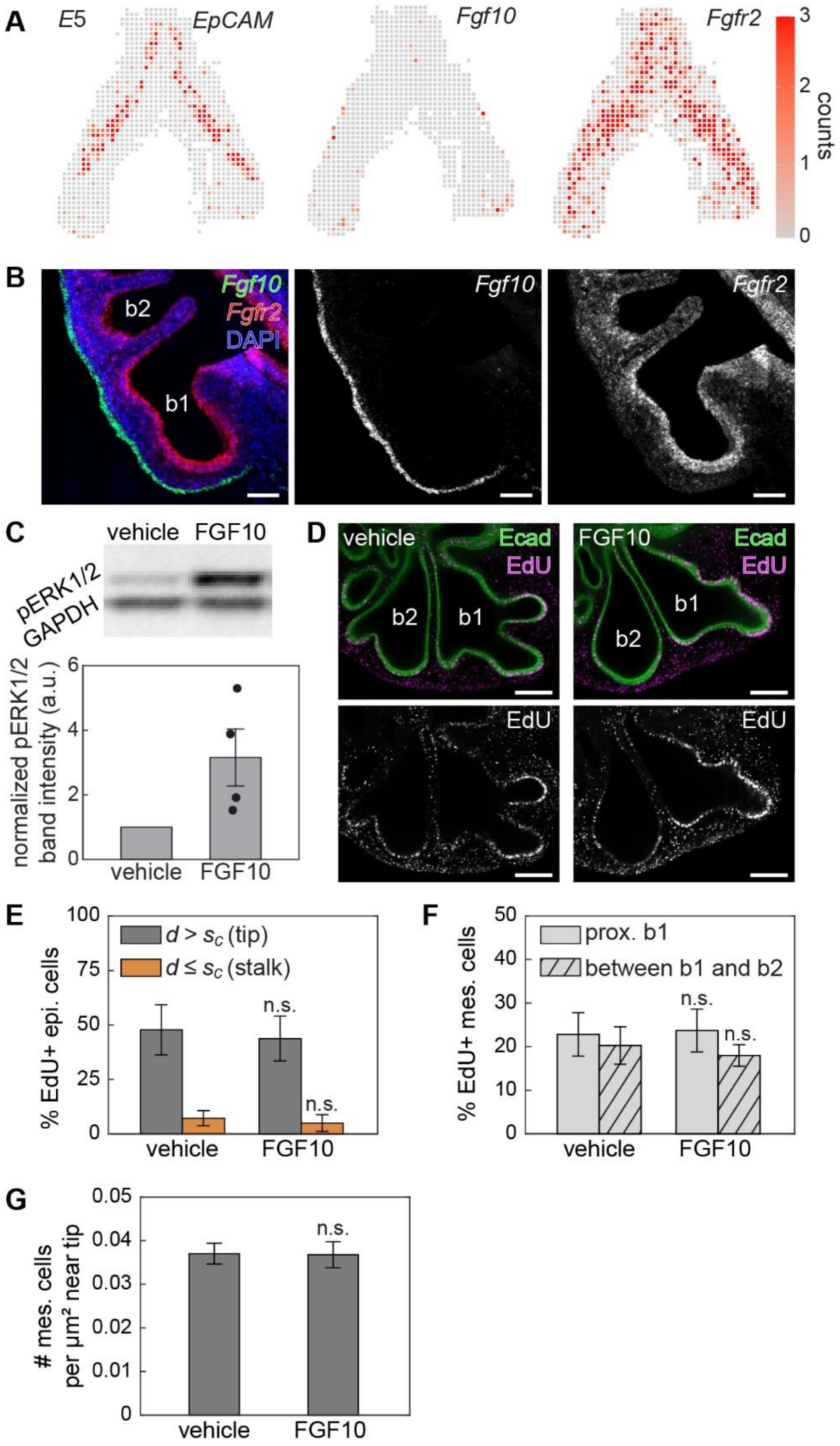

Fig. S3. Exogeneous addition of FGF10 has no effect on the spatial pattern of proliferation in the embryonic lung – related to Fig. 3. (**A**) Representative gene-expression patterns of *EpCAM*, which marks the epithelium, *Fgf10,* and *Fgfr2*. (**B**) In situ hybridization analysis for *Fgf10* and *Fgfr2* in *E*6 lungs cultured for 24 hours. Scale bars, 100 µm. (**C**) Representative immunoblots for pERK1/2 and GAPDH in lungs cultured with FGF10 or vehicle control, and graph showing the normalized intensity of the pERK1/2 band from 4 replicates. (**D**)

bioRxiv preprint doi: https://doi.org/10.64898/2026.03.16.712215; this version posted March 18, 2026. The copyright holder for this preprint (which was not certified by peer review) is the author/funder, who has granted bioRxiv a license to display the preprint in perpetuity. It is made available under aCC-BY 4.0 International license.

Fluorescence images of EdU incorporation (magenta) in lung explants cultured with exogeneous FGF10 or vehicle control, counterstained for E-cadherin (green). Scale bars, 100 µm. (**E**) Graph showing the percentage of EdU-positive cells in the epithelium with and without exogenous FGF10. Shown are the average and s.d. of 3 lungs per condition. (**F**) Graph of percentage of EdU-positive cells in the mesenchyme in lungs cultured with FGF10 or vehicle control. Solid and hatched bars denote percentage of EdU-positive mesenchymal cells proximal to b1 and in between b1 and b2, respectively. n.s. stands for not significant. (**G**) Graph showing the density of mesenchymal cells near the tip in lung explants cultured with exogenous FGF10 or vehicle control. Shown are the average and s.d. of 3 lungs per condition.

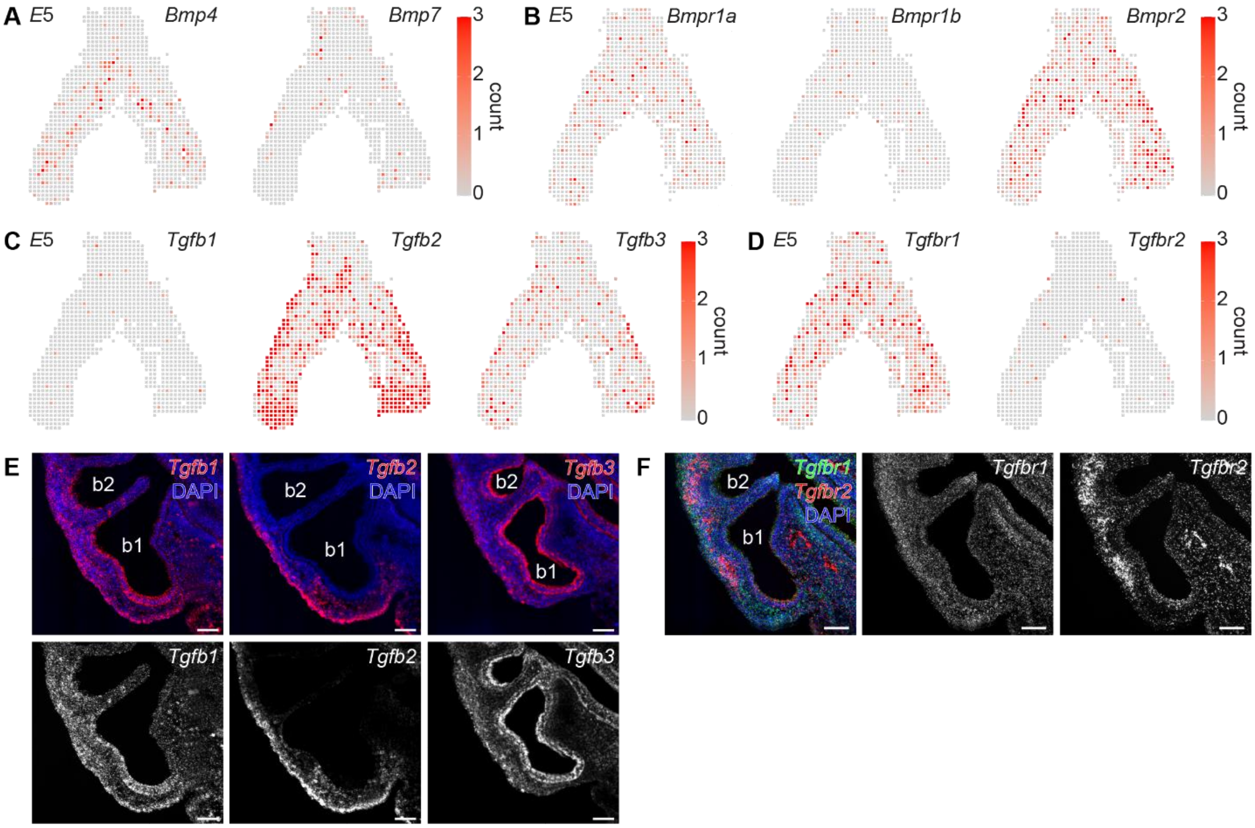

Fig. S4. Spatial patterns of expression of inhibitory morphogens for epithelial cells and their receptors in the developing lung – related to Fig. 3. (**A**) Representative gene-expression patterns of *Bmp4* and *Bmp7*. (**B**) Representative gene-expression patterns of *Bmpr1a*, *Bmpr1b*, and *Bmpr2*. (**C**) Representative gene-expression patterns of *Tgfb1*, *Tgfb2*, and *Tgfb3*. (**D**) Representative gene-expression patterns of *Tgfbr1* and *Tgfbr2*. (**E**) Fluorescence in situ hybridization for *Tgfb1*, *Tgfb2*, and *Tgfb3* in *E*6 lungs cultured for 24 hours. Scale bars, 100 µm. (**F**) Fluorescence in situ hybridization for *Tgfbr1* and *Tgfbr2* in *E*6 lungs cultured for 24 hours. Scale bars, 100 µm.

bioRxiv preprint doi: https://doi.org/10.64898/2026.03.16.712215; this version posted March 18, 2026. The copyright holder for this preprint (which was not certified by peer review) is the author/funder, who has granted bioRxiv a license to display the preprint in perpetuity. It is made available under aCC-BY 4.0 International license.

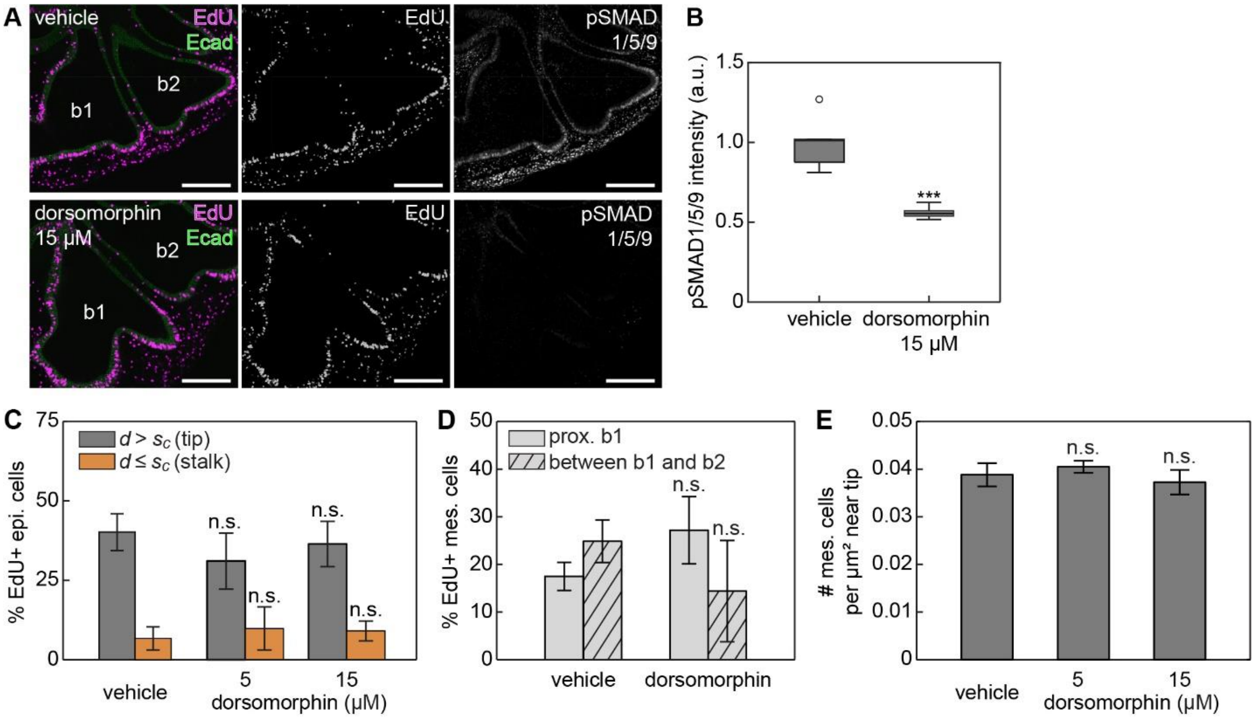

Fig. S5. Inhibiting signaling from BMP has no effect on the spatial patterns of proliferation in the embryonic lung – related to Fig. 3. (**A**) Fluorescence images of EdU incorporation (magenta) and pSMAD1/5/9 staining in lung explants cultured with BMP receptor inhibitor, dorsomorphin, or vehicle control, counterstained for E-cadherin (green). Scale bars, 100 µm. (**B**) Graph showing the relative intensity of pSMAD1/5/9 staining in lung explants cultured with dorsomorphin or vehicle control. Shown are the average and s.d. of 3 lungs per condition. ***p<0.001 (**C**) Graph showing the percentage of EdU-positive cells in the epithelium of lung explants cultured with dorsomorphin or vehicle control. Shown are the average and s.d. of 6 lungs per condition. n.s. stands for not significant. (**D**) Graph showing the percentage of EdU-positive cells in the mesenchyme in lungs cultured with dorsomorphin or vehicle control. Solid and hatched bars respectively denote percentage of EdU-positive mesenchymal cells proximal to b1 and in between b1 and b2. n.s. stands for not significant. (**E**) Graph showing the density of mesenchymal cells near the tip in lung explants cultured with dorsomorphin or vehicle control. Shown are the average and s.d. of 3 lungs per condition.

bioRxiv preprint doi: https://doi.org/10.64898/2026.03.16.712215; this version posted March 18, 2026. The copyright holder for this preprint (which was not certified by peer review) is the author/funder, who has granted bioRxiv a license to display the preprint in perpetuity. It is made available under aCC-BY 4.0 International license.

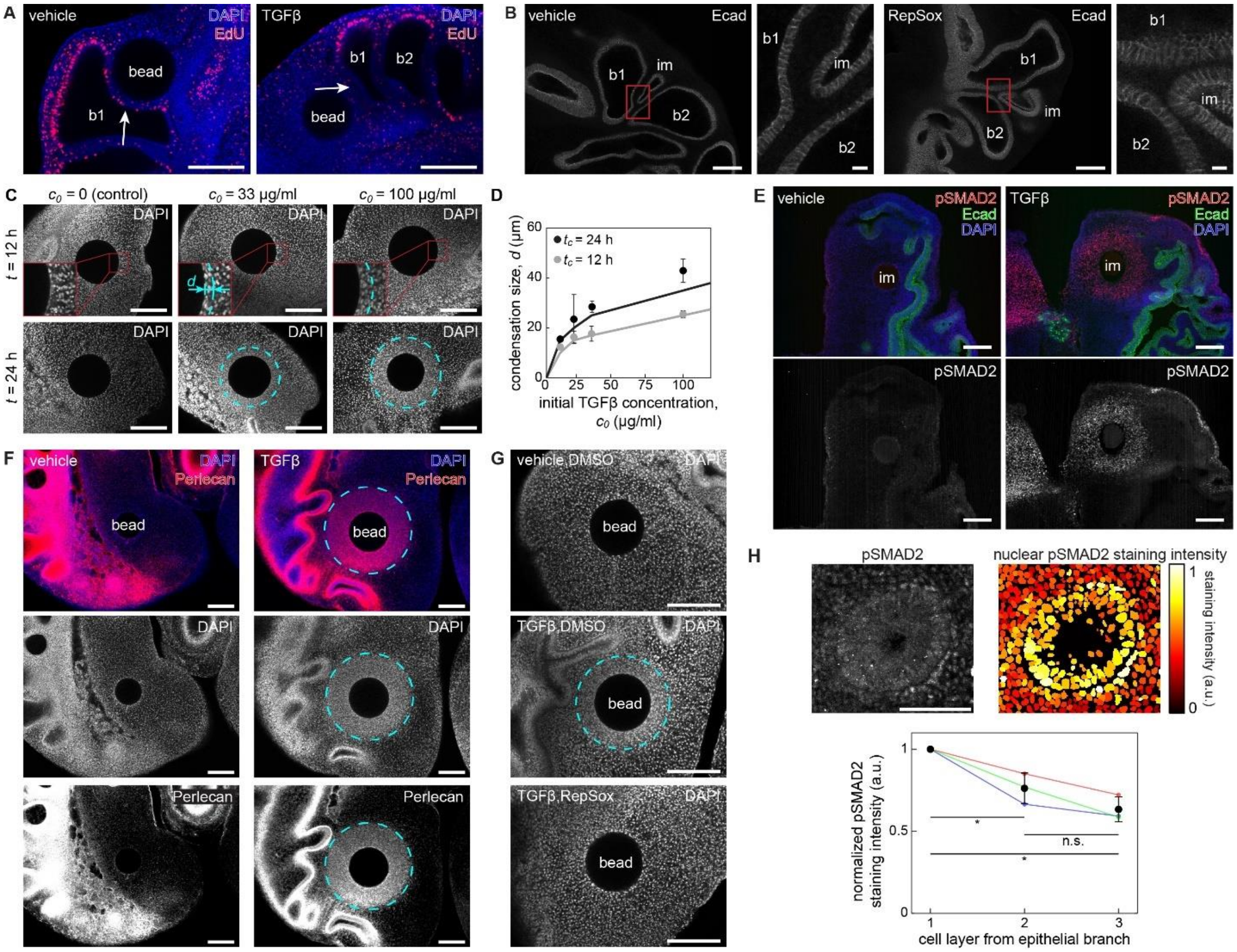

Fig. S6. Effects of TGFβ on epithelial and mesenchymal cells in the developing lung – related to Fig. 4. (**A**) Fluorescence images of nuclei (blue) and EdU incorporation (red) in lung explants inserted with TGFβ-containing bead or vehicle control. The beads were inserted proximal to b1. In contrast to control, few EdU-positive mesenchymal cells are observed in TGFβ-containing bead-inserted lung. White arrows denote proximal stalk of b1. (**B**) Fluorescence images of epithelium in branch-implanted lung explants treated with RepSox or vehicle control. The distance between implanted and native branches is small in RepSox-treated lungs. Scale bars, 100 μm and 10 μm (inset). (**C**) Fluorescence images of nuclei in lung explants cultured for 12- or 24-hours after implantation of beads with different TGFβ concentrations. (**D**) Graph showing the sizes of condensations in bead-implanted lung explants with different initial TGFβ concentration and culture time. (**E**) Fluorescence images of nuclei (blue) and pSMAD2 (red) counterstained for E-cadherin (green). Strong circular pSMAD2 staining is observed near TGFβ-containing beads. (**F**) Fluorescence images of nuclei (blue) and perlecan (red) in lung explants inserted with TGFβ-containing bead or vehicle control. Strong perlecan staining is observed in mesenchymal condensations near TGFβ-containing beads. (**G**) Fluorescence images of nuclei in lung explants cultured with or without RepSox after being inserted with TGFβ-containing beads or vehicle control. Mesenchymal condensations are only observed in lungs inserted with TGFβ-containing beads and cultured without RepSox. (**H**) pSMAD2 staining and nuclear-masked pSMAD2 staining intensity near the branch. Scale bar, 50 μm. Subepithelial

bioRxiv preprint doi: https://doi.org/10.64898/2026.03.16.712215; this version posted March 18, 2026. The copyright holder for this preprint (which was not certified by peer review) is the author/funder, who has granted bioRxiv a license to display the preprint in perpetuity. It is made available under aCC-BY 4.0 International license.

mesenchymal cells exhibit strong pSMAD2 staining. Cyan dotted lines denote condensations (**D, F, G**). Scale bars, 100 μm (**A, C, E, F, G**).

bioRxiv preprint doi: https://doi.org/10.64898/2026.03.16.712215; this version posted March 18, 2026. The copyright holder for this preprint (which was not certified by peer review) is the author/funder, who has granted bioRxiv a license to display the preprint in perpetuity. It is made available under aCC-BY 4.0 International license.

Captions for Supplementary Videos

Supplementary Video S1 – related to Fig. 2. Live-imaging of cytoplasmic-GFP expressing *E*5 chicken lung. Duration = 20 h. Scale bar, 50 µm.

Supplementary Video S2 – related to Fig. 5. Live-imaging of cytoplasmic-GFP expressing *E*6 chicken lung implanted with vehicle-containing bead. Duration = 16 h. Scale bar, 50 µm

Supplementary Video S3 – related to Fig. 5. Live-imaging of cytoplasmic-GFP expressing *E*6 chicken lung implanted with TGFβ-containing bead. Duration = 16 h. Scale bar, 50 µm
